# Comparative evaluation of diffusion models for detecting gray matter microstructural alterations in post-stroke depression patients

**DOI:** 10.3389/fneur.2026.1831557

**Published:** 2026-09-30

**Authors:** Fang Zhang, Jing Zhang, Lei Zhang, Hengjun Jin, Daqing Li, Wei Zhao

**Affiliations:** Department of Radiology, Huaibei People's Hospital, Huaibei, Anhui, China

**Keywords:** diffusion kurtosis imaging, diffusion tensor imaging, gray matter microstructural alterations, mean apparent propagator, neurite orientation dispersion and density imaging, post-stroke depression

## Abstract

**Background:**

Post-stroke depression (PSD) is often underdiagnosed because its gray matter microstructural abnormalities can be subtle. This study compared four diffusion models (DTI, DKI, MAP, and NODDI) to identify objective imaging biomarkers for the early detection of PSD.

**Methods:**

Sixty-four participants were enrolled, including 23 age- and sex-matched healthy controls (HC), 26 patients with acute cerebral infarction without PSD (non-PSD; HAMD-21 < 7), and 15 patients with PSD (HAMD-21 ≥ 7). Clinical assessments (HAMD-21, ADL, MoCA, and MMSE) and diffusion spectrum imaging were performed. Intergroup differences in gray matter microstructure, diagnostic performance based on the area under the curve (AUC), and partial correlations adjusted for age, sex, and years of education were analyzed. Infarct location was not included as a covariate.

**Results:**

Significant differences among the three groups were observed in age, years of education, and all clinical scale scores (*p* < 0.05). Altered gray matter microstructure was primarily identified in the middle frontal gyrus, posterior cingulate gyrus, paracingulate gyrus and amygdala. For group discrimination, the left anterior cingulate DKI MD_p10 demonstrated the best performance for distinguishing HC from non-PSD (AUC = 0.916). The left putamen NODDI ODI_p10 showed the highest AUC (0.916) for distinguishing HC from PSD, whereas the left putamen NODDI ODI_p50 yielded the highest AUC (0.856) for distinguishing non-PSD from PSD. After FDR correction, multiple diffusion metrics were significantly correlated with MMSE and MoCA scores, whereas no imaging parameter showed a significant correlation with HAMD-21.

**Conclusion:**

Multimodal diffusion MRI detects microstructural abnormalities within emotion- and cognition-related circuits, particularly the amygdala and cingulate cortex, across the three groups. Temporolimbic NODDI parameters showed relatively higher AUC values in distinguishing PSD from HC, whereas anterior cingulate DKI metrics showed relatively higher AUC values in differentiating non-PSD stroke patients from HC. Combining multiple diffusion models may aid in the identification of PSD; however, these findings require further validation because infarct location was not adjusted for in the analyses.

## Highlights

- Multimodal diffusion MRI detects gray matter microstructural changes in PSD.
- NODDI metrics show potential for distinguishing PSD from HC.
- DKI metrics help differentiate non-PSD stroke patients from HC.
- Diffusion biomarkers correlate with cognitive scores, not depression severity.

## Introduction

Stroke remains a major public health challenge in China, characterized by high incidence, mortality, and disability rates, imposing substantial burdens on patients, families, and healthcare systems (1). Among the myriad complications following stroke, post-stroke depression (PSD) is particularly prevalent and insidious. Epidemiological studies indicate that approximately one-third of stroke survivors develop PSD, which can arise at any stage post-stroke, from the acute phase to months or even years after the initial event (2, 3). This high prevalence highlights the urgent need for targeted research, as PSD’s consequences extend beyond psychological distress. Clinically, PSD presents a complex constellation of symptoms, including persistent low mood, anhedonia, psychomotor retardation, irritability, and, in severe cases, suicidal ideation (4). These symptoms not only exacerbate psychological suffering but also impede neurological recovery. PSD is consistently associated with poorer functional outcomes, higher rates of recurrent vascular events, and increased mortality compared with non-depressed stroke patients (4). For example, a five-year follow-up study in a Chinese cohort demonstrated that persistent PSD independently predicts reduced quality of life, highlighting the long-term impact of unrecognized or untreated PSD (1). Despite its clinical significance, PSD remains frequently underdiagnosed, partly due to the absence of objective, imaging-based biomarkers that can complement subjective clinical assessments.

The pathophysiology of PSD is increasingly linked to structural and functional abnormalities in neural circuits regulating emotion. In particular, the limbic–cortical-striatal-pallidal-thalamic (LCSPT) circuit has emerged as a critical focus (5). This circuit integrates cortical regions, including the prefrontal cortex and cingulate gyrus, with subcortical structures such as the amygdala and thalamus, thereby modulating emotional responses. Stroke-induced lesions, whether directly via infarction or indirectly via disruption of axonal connections, can impair this circuit, leading to dysfunctional emotion processing. Gray matter microstructural abnormalities within these regions are hypothesized to be a key underlying mechanism (6). Previous research on PSD has primarily focused on white matter integrity, emphasizing inter-regional connectivity, while placing less attention on gray matter, where neuronal cell bodies, dendrites, and synapses, the core substrates of local information processing, are concentrated. Examining gray matter microstructure provides unique value, as it directly reflects alterations such as neurite organization and cellular density in regions crucial for emotion and cognition (amygdala and anterior cingulate gyrus). These regions are more closely associated with PSD’s core symptoms, including emotional dysregulation and cognitive decline. Compared with subjective clinical scales or white matter assessments, advanced diffusion magnetic resonance imaging (MRI) models, diffusion tensor imaging (DTI), diffusion kurtosis imaging (DKI), mean apparent propagator (MAP), and neurite orientation dispersion and density imaging (NODDI), enable objective quantification of subtle gray matter changes. This approach reduces subjective bias, detects pre-clinical pathological alterations, and offers more sensitive and specific biomarkers for early PSD identification and mechanistic insights.

Diffusion MRI (dMRI) has become an essential tool for investigating such microstructural changes. DTI, the most established method, quantifies directional water diffusion to assess tissue integrity, with parameters such as fractional anisotropy (FA) and mean diffusivity (MD) reflecting white matter tract coherence (7). However, DTI is limited by its assumption of Gaussian diffusion, which inadequately captures the complexity of gray matter microstructure. DKI overcomes this limitation by modeling non-Gaussian diffusion, providing metrics such as mean kurtosis (MK) that yield deeper insights into cellular density and tissue heterogeneity (8, 9). More recently, NODDI has emerged as a powerful approach for quantifying neurite morphology at the microscale. Parameters such as neurite orientation dispersion index (ODI) and intracellular volume fraction (ICVF) characterize the arrangement and density of axons and dendrites (10, 11). Complementing these, MAP imaging provides unique metrics that capture how water movement diffusion interacts with cellular compartments, enabling a more detailed characterization of gray matter microstructural alterations than single-dimension diffusion assessments, including quasi-intracellular volume (QIV) and radial tissue oriented part (RTOP), which describe water diffusion patterns in relation to cellular compartments (11).

Although individual studies have applied these models to stroke or depression, few have systematically compared their diagnostic efficacy in detecting PSD-specific gray matter alterations. While DTI, DKI, MAP, and NODDI have each been used in neuropsychiatric research, their relative diagnostic value in post-stroke depression remains unclear. Determining which model is most sensitive for detecting PSD-related gray matter changes is critical for advancing both research and clinical practice. This study represents the first head-to-head comparison of advanced diffusion models in PSD, addressing this gap by directly evaluating DTI, DKI, MAP, and NODDI to identify the most sensitive and informative imaging markers for early and objective PSD detection.

## Methods

### Study subjects

A total of 64 participants were enrolled in this study at Huaibei People’s Hospital between March 2024 and April 2025. The sample included 23 healthy controls (HC) and 26 non-PSD patients with acute cerebral infarction (HAMD-21 < 7), and 15 PSD patients with acute cerebral infarction (HAMD-21 ≥ 7). HC were strictly matched to stroke patients by age and sex, and were required to have no history of vascular risk factors (e.g., hypertension, diabetes mellitus, and dyslipidemia) in order to minimize potential confounding effects of vascular pathology on gray matter microstructural assessments. All stroke patients were initially enrolled as a single cohort and matched with HC by age and sex at recruitment. Two weeks after admission, the stroke cohort was stratified into PSD and non-PSD groups according to HAMD-21 scores, resulting in slight age differences among the three final groups. Age, sex and years of education were included as covariates in all statistical analyses to minimize potential confounding. The sample size of 64 (23 HC, 26 non-PSD, 15 PSD) was determined based on prior literature in PSD neuroimaging research (12, 13). All participants underwent comprehensive clinical assessments and MRI scanning. Inclusion criteria were: first-time stroke confirmed by CT or MRI and the absence of severe communication or cognitive impairments. Exclusion criteria included any prior psychiatric disorder or contraindications to MRI. The study protocol was approved by the institutional ethics committee, and written informed consent was obtained from all participants.

Clinical evaluations were conducted 2 weeks after admission and included the Mini-Mental State Examination (MMSE), Montreal Cognitive Assessment (MoCA), Activities of Daily Living (ADL), and Hamilton Depression Rating Scale-21 (HAMD-21). Based on HAMD-21 scores, patients were classified into the PSD group (HAMD-21 ≥ 7, *n* = 15) or the non-PSD group (HAMD-21 < 7, *n* = 26).

### MRI acquisition

Imaging was performed on a 3.0 Tesla MAGNETOM VIDA scanner (Siemens Healthineers, Erlangen, Germany). The scanning protocol included high-resolution structural and diffusion imaging sequences.

High-resolution T1-weighted structural images were acquired using a 3D SPACE sequence (TR = 2,200 ms, TE = 2.46 ms, field of view [FOV] = 230 × 230 mm^2^, voxel size = 0.9 × 0.9 × 0.9 mm^3^, 208 slices). The scan time for this sequence was 5 min 21 s. T2-weighted images were obtained in transverse and sagittal planes (TR = 4,000 ms, TE = 107 ms, FOV = 240 × 240 mm^2^, voxel size = 0.6 × 0.6 × 5 mm^3^, 18 slices), with a scan time of 45 s.

Diffusion spectrum imaging (DSI) was acquired using a transverse sequence (TR = 2,900 ms, TE = 116 ms, FOV = 220 × 220 mm^2^, voxel size = 0.8 × 0.8 × 2.2 mm^3^, 60 slices). The acquisition employed a q4 semi-sampling scheme, which involved radial q-space sampling on a hemisphere with a radius of 4, covering b-values ranging from 0 to 3,000 s/mm^2^. Specifically, the sequence included 128 diffusion-weighted volumes acquired at multiple b-values (distributed across the range 0–3,000 s/mm^2^) and one b = 0 image, parallel imaging (GeneRalized Autocalibrating Partial Parallel Acquisition) acceleration factor = 2. Phase encoding was performed in the anterior–posterior direction. The total scan time was 6 min 54 s. See Table 1 for detailed MRI acquisition parameters. No susceptibility-distortion correction was applied to the diffusion-weighted images.

**Table 1:** Imaging parameters.

Parameter	T1 MPRAGE	T2 sag/tra	DSI
TR (ms)	2,200	4,000	2,900
TE (ms)	2.46	107	116
FOV (mm × mm)	230 × 230	240 × 240	220 × 220
Resolution (mm^3^)	0.9 × 0.9 × 0.9	0.6 × 0.6 × 5	0.8 × 0.8 × 2.2
Slice	208	18	60
Slice Thickness (mm)	0.9	5	2.2
*B*-value	/	/	0–3,000
Scan time	5 min 21 s	45 s	6 min 54 s

### Data preprocessing and post-processing

Image preprocessing and quantitative analysis were performed using NeuDiLab software (version 1.0, Chengdu Zhongying Medical Technology Co., Ltd., China) developed in-house based on the open-resource tool DIPY (Diffusion Imaging in Python, http://nipy.org/dipy). First, the participant’s DSI data were converted from DICOM format to NIfTI format using dcm2nii GUI, and the corresponding bvecs and bvals files were generated. Preprocessing, including correction for head motion and eddy currents and skull stripping, was conducted using NeuDiLab software. No susceptibility-distortion correction was applied to the diffusion-weighted images. The DSI-derived parameters were then calculated for subsequent quantitative analysis and quality control. Diffusion-derived parameters were extracted across multiple models: for DTI, axial diffusivity (AD), FA, mean diffusivity (MD), and radial diffusivity (RD); for DKI, MD and MK; for MAP, QIV, return-to-axis probability (RTAP), RTOP, and return-to-plane probability (RTPP); and for NODDI, extracellular volume fraction (ECVF), ICVF, isotropic volume fraction (ISOVF), and ODI (14–16). Details regarding the model fitting methodology are provided in the Supplementary materials. All images were spatially normalized to the Montreal Neurological Institute (MNI) template using SPM, and 116 brain regions were segmented based on the Automated Anatomical Labeling (AAL) atlas. To facilitate accurate analysis of gray matter microstructure, pre-defined regions of interest (ROIs) were selected, including the bilateral middle frontal gyri, anterior cingulate cortices, posterior cingulate cortices, hippocampi, parahippocampal gyri, amygdalae, superior occipital gyri, superior parietal gyri, inferior parietal gyri, caudate nuclei, putamina, globus pallidi, thalami, and middle temporal gyri (17). For each region of interest, the mean and standard deviation of all parameters were calculated. First, atlas-based ROI definition, followed by individual visual inspection to confirm no lesion overlap. All ROIs were visually inspected on each participant’s T1-weighted and diffusion-weighted images to exclude gross, macroscopically visible overlap ischemic lesions. The majority of infarcts in our cohort were located in the deep gray matter and white matter, whereas the ROIs of interest were situated in the limbic cortex and amygdala, which are anatomically distinct from the predominant infarct territories. No gross macroscopic overlap between target ROIs and overt infarct lesions was observed.

### Statistical analysis

Statistical analyses were conducted using SPSSAU and SPSS version 19.0 (IBM Corp., Armonk, NY, United States). The Shapiro–Wilk test was first used to assess the normality of all multimodal diffusion metrics and clinical scale variables. Normally distributed variables with homogeneous variance were analyzed using one-way analysis of covariance (ANCOVA), with age, sex and years of education included as covariates. For variables that did not meet the assumptions of normality, the effects of age, sex and education were first removed using linear regression, and the resulting residuals were subsequently analyzed using Kruskal–Wallis *H* tests. Benjamini–Hochberg false discovery rate (BH-FDR) correction was applied to all omnibus *p* values from the ANCOVA and Kruskal–Wallis tests. Only imaging metrics with a global FDR-adjusted *q* < 0.05 were retained for *post hoc* pairwise comparisons. Tukey’s honestly significant difference (HSD) test was used for pairwise comparisons of normally distributed variables, whereas pairwise Mann–Whitney U tests with BH-FDR correction were used for non-normally distributed data. Partial Spearman rank correlations, adjusted for age, sex, and years of education were then performed between the screened significant ROI-based diffusion metrics and continuous MMSE, MoCA and HAMD-21 scores. BH-FDR correction was performed independently within each clinical scale. Imaging biomarkers that simultaneously met the criteria FDR-adjusted *q* < 0.05 and an absolute partial correlation coefficient |ρ| ≥ 0.3 were subsequently included in receiver operating characteristic (ROC) curve analyses. For each pairwise group comparison (HC vs. non-PSD, HC vs. PSD, and non-PSD vs. PSD), the 10 biomarkers with the highest area under the curve (AUC) values were selected and plotted separately. A two-sided corrected *P* < 0.05 was considered statistically significant for all statistical analyses.

## Results

### Demographic and clinical characteristics

As summarized in Table 2, significant differences in age (*p* = 0.02), education level (*p* = 0.02), HAMD-21 (*p* < 0.001), ADL (*p* < 0.001), MoCA (*p* < 0.001), and MMSE (*p* < 0.001) were observed among groups. Compared with non-PSD patients and HC, PSD patients exhibited higher HAMD-21 scores, lower education levels, and greater impairment in activities of daily living. No significant differences were found in gender distribution, body mass index (BMI), or insomnia status across groups (*p* > 0.05). Consistent with their established diagnostic value, both MoCA and MMSE demonstrated strong discriminative power among groups (MoCA: *p* < 0.001; MMSE: *p* < 0.001). An obvious gradient of cognitive performance was observed for MoCA, with HC achieving the highest scores, followed by non-PSD patients, and PSD patients scoring lowest. For MMSE, HC had the highest scores, but non-PSD patients scored lower than PSD patients, indicating a non-monotonic pattern across groups. The full per-patient lesion distribution is provided in Supplementary Table S1.

**Table 2:** Demographic and clinical characteristics of patients and HC.

Characteristic	Type			*p*
	HC	Non_PSD	PSD	
Age	58.39 ± 9.08	63.27 ± 9.08	54.93 ± 8.75	0.02
Female	10.00 (43.48%)	10.00 (38.46%)	4.00 (26.67%)	0.97
Height	1.66 ± 0.09	1.65 ± 0.17	1.65 ± 0.08	0.97
Weight	66.74 ± 10.27	69.04 ± 8.60	68.20 ± 6.75	0.66
BMI	24.52 ± 2.68	25.41 ± 2.85	23.66 ± 2.81	0.24
Level of education	8.90 ± 5.13	7.80 ± 2.10	5.40 ± 4.65	0.02
MoCA	28.00 (27.00, 30.00)	18.00 (15.00, 20.00)	16.80 (13.00, 18.00)	<0.001
HAMD-21	3.43 ± 2.11	4.27 ± 1.16	14.47 ± 3.43	<0.001
ADL	100.00 (100.00, 100.00)	100.00 (100.00, 100.00)	90.00 (80.00, 100.00)	<0.001
MMSE	30.00 (29.00, 30.00)	20.0 (18.00, 23.00)	21.0 (20.00, 27.00)	<0.001

PSD, post-stroke depression; MoCA, Montreal Cognitive Assessment; MMSE, Mini-Mental State Examination; HAMD-21, Hamilton Depression; ADL, Activities of Daily Living; BMI, body mass index.

### Identification of regions of interest (ROIs)

Among the predefined ROIs, group comparisons revealed significant microstructural alterations in the middle frontal gyrus, posterior cingulate gyrus, paracingulate gyrus and amygdala. These regions were therefore retained for further analysis.

### Diagnostic efficacy

#### HC vs. non-PSD

For discrimination between the HC and non-PSD groups, the top 10 imaging biomarkers yielded AUC values ranging from 0.890 to 0.916 (Figure 1A and Table 3). The left anterior cingulate DKI MD_p10 demonstrated the highest diagnostic efficacy (AUC = 0.916), followed by the right hippocampus DKI MD_p10 (AUC = 0.908). Other high-performance markers included the right amygdala DTI MD_p25 (AUC = 0.906), left anterior cingulate DKI MD_p25 (AUC = 0.905) and left inferior parietal DTI RD_p10 (AUC = 0.903). All selected diffusion metrics exhibited good discriminative performance, with all AUC values exceeding 0.89. These findings suggest that microstructural alterations in frontal, limbic, parietal, and occipital gray matter may effectively distinguish HC from stroke survivors without post-stroke depression.

**Figure 1:**
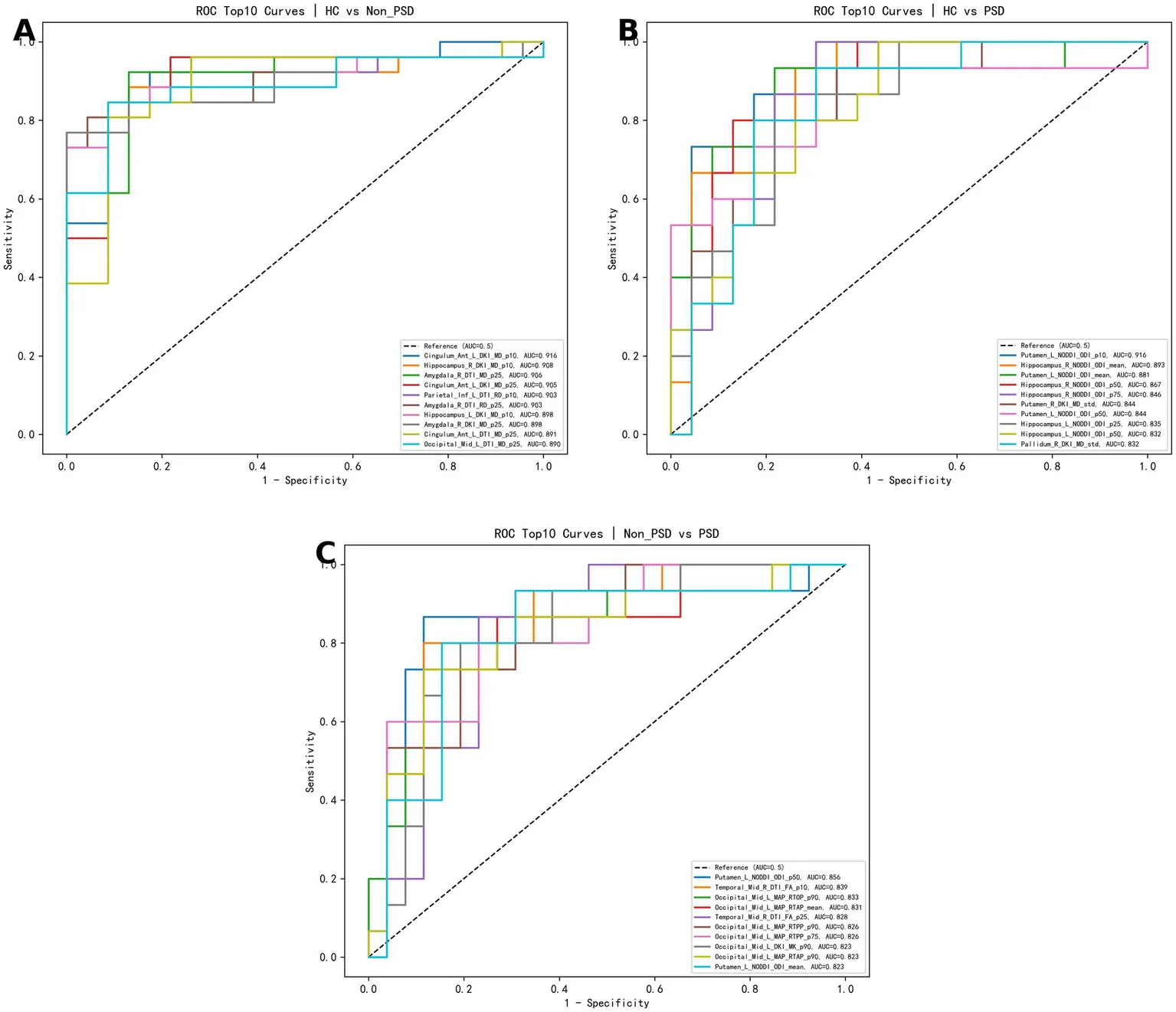
ROC curves of the top 10 screened multimodal diffusion imaging biomarkers for three pairwise group comparisons. Biomarkers satisfied dual screening criteria: FDR-adjusted *q* < 0.05 and |partial Spearman correlation coefficient| ≥ 0.3 with MMSE or MoCA scores. **(A)** HC versus non-PSD; **(B)** HC versus PSD; **(C)** non-PSD versus PSD. The black dashed line represents the reference line with AUC = 0.5. Each colored curve corresponds to one AAL gray matter region-derived diffusion metric, annotated with brain region name and AUC value. HC, healthy controls; PSD, post-stroke depression; ROC, receiver operating characteristic; AUC, area under the curve.

**Table 3:** ROC analysis results of top 10 ROI regions (ranked by AUC in descending order) of HC and Non_PSD patients.

Parameters	AUC (95% CI)	Cut off value	Sensitivity	Specificity
Cingulum_Ant_L_DKI_MD_p10	0.916 (0.765–1.0)	795.99	0.9231	0.8261
Hippocampus_R_DKI_MD_p10	0.908 (0.743–1.0)	868.67	0.8462	0.9130
Amygdala_R_DTI_MD_p25	0.906 (0.739–1.0)	712.52	0.9231	0.8696
Cingulum_Ant_L_DKI_MD_p25	0.905 (0.734–1.0)	882.53	0.9615	0.7826
Parietal_Inf_L_DTI_RD_p10	0.903 (0.730–1.0)	599.47	0.7692	1.000
Amygdala_R_DTI_RD_p25	0.903 (0.730–1.0)	624.94	0.8077	0.9565
Amygdala_R_DKI_MD_p25	0.898 (0.717–1.0)	886.21	0.7692	1.0000
Hippocampus_L_DKI_MD_p10	0.898 (0.717–1.0)	871.58	0.7308	1.0000
Cingulum_Ant_L_DTI_MD_p25	0.891 (0.700–1.0)	747.88	0.8077	0.9130
Occipital_Mid_L_DTI_MD_p25	0.890 (0.695–1.0)	752.12	0.8462	0.9130

#### HC vs. PSD

For discrimination between the HC and PSD groups, the AUC values of the top 10 imaging biomarkers ranged from 0.832 to 0.916 (Figure 1B and Table 4). The left putamen NODDI ODI_p10 exhibited the best diagnostic performance (AUC = 0.916). The second-best-performing biomarker was the right hippocampus NODDI ODI_mean (AUC = 0.893), followed by the left putamen NODDI ODI_mean (AUC = 0.881). Consistent with the intergroup comparison results, most of the best-performing biomarkers were NODDI ODI measures located in limbic and temporal regions. All selected imaging features achieved AUC value >0.83. Among them, NODDI-derived neurite dispersion metrics in the medial temporal lobe show high potential for distinguishing HC from PSD patients.

**Table 4:** ROC analysis results of top 10 ROI regions (ranked by AUC in descending order) of HC and PSD patients.

Parameters	AUC (95% CI)	Cut off value	Sensitivity	Specificity
Putamen_L_NODDI_ODI_p10	0.916 (0.764–1.0)	325.01	0.8667	0.8261
Hippocampus_R_NODDI_ODI_mean	0.893 (0.703–1.0)	547.07	0.9333	0.7391
Putamen_L_NODDI_ODI_mean	0.881 (0.674–1.0)	554.85	0.9333	0.7826
Hippocampus_R_NODDI_ODI_p50	0.867 (0.638–1.0)	586.86	0.8000	0.8696
Hippocampus_R_NODDI_ODI_p75	0.846 (0.588–1.0)	655.40	1.0000	0.6957
Putamen_R_DKI_MD_std	0.844 (0.581–1.0)	282.46	0.9333	0.6522
Putamen_L_NODDI_ODI_p50	0.844 (0.581–1.0)	575.11	0.9333	0.6957
Hippocampus_L_NODDI_ODI_p25	0.835 (0.560–1.0)	494.04	0.8000	0.7826
Hippocampus_L_NODDI_ODI_p50	0.832 (0.553–1.0)	576.15	1.0000	0.5652
Pallidum_R_DKI_MD_std	0.832 (0.553–1.0)	479.55	0.9333	0.6957

#### Non-PSD vs. PSD

For discrimination between stroke patients with and without PSD, the top 10 imaging biomarkers yielded AUC values ranging from 0.823 to 0.856 (Figure 1C and Table 5). The left putamen NODDI ODI_p50 demonstrated the highest diagnostic performance (AUC = 0.856). The second-best-performing biomarker was the right middle temporal gyrus DTI FA_p10 (AUC = 0.839), followed by the left middle occipital lobe MAP RTOP_p90 (AUC = 0.833). The candidate biomarkers comprised multimodal diffusion metrics derived from NODDI, DTI, and MAP and were distributed across the putamen, middle temporal gyrus and occipital lobe. All selected features yielded AUC values >0.82, suggesting that multimodal gray matter microstructural metrics may effectively distinguish stroke survivors with PSD from those without depressive symptoms.

**Table 5:** ROC analysis results of top 10 ROI regions (ranked by AUC in descending order) of Non_PSD and PSD patients.

Parameters	AUC (95% CI)	Cut off value	Sensitivity	Specificity
Putamen_L_NODDI_ODI_p50	0.856 (0.612–1.0)	589.90	0.8667	0.8846
Temporal_Mid_R_DTI_FA_p10	0.839 (0.569–1.0)	78.31	0.8000	0.8846
Occipital_Mid_L_MAP_RTOP_p90	0.833 (0.557–1.0)	636.98	0.7333	0.8846
Occipital_Mid_L_MAP_RTAP_mean	0.831 (0.551–1.0)	603.98	0.7333	0.8846
Temporal_Mid_R_DTI_FA_p25	0.828 (0.544–1.0)	100.60	0.8667	0.7692
Occipital_Mid_L_MAP_RTPP_p75	0.826 (0.538–1.0)	664.52	0.8000	0.7692
Occipital_Mid_L_MAP_RTPP_p90	0.826 (0.538–1.0)	634.85	0.7333	0.8077
Occipital_Mid_L_DKI_MK_p90	0.823 (0.532–1.0)	1032.20	0.8000	0.8077
Putamen_L_NODDI_ODI_mean	0.823 (0.532–1.0)	809.93	0.8000	0.8462
Occipital_Mid_L_MAP_RTAP_p90	0.823 (0.532–1.0)	561.71	0.7333	0.8846

### Correlation analysis

Partial Spearman rank correlations adjusted for age, sex and years of education were calculated for all FDR-screened significant AAL-based gray matter diffusion metrics. Independent BH-FDR correction was performed separately for MMSE, MoCA and HAMD-21. Multiple multimodal diffusion metrics met the prespecified criteria of FDR *q* < 0.05 and |ρ| ≥ 0.3 for associations with MMSE and MoCA. The four markers showing the strongest associations with MMSE and the four showing the strongest associations with MoCA, ranked by the absolute value of the partial correlation coefficient, were visualized using covariate-adjusted residual scatter plots with fitted trend lines (Figures 2, 3). For MMSE, right anterior cingulate DKI MD_mean showed a strong negative correlation with MMSE (*ρ* = −0.583, *q* < 0.001). Positive correlations were observed for left parahippocampal MAP RTOP_p10 (*ρ* = 0.569, *q* < 0.001) and right middle temporal pole DTI FA_p10 (*ρ* = 0.565, *q* < 0.001), whereas left supramarginal gyrus DTI RD_p75 was negatively correlated with MMSE (*ρ* = −0.560, *q* < 0.001). For MoCA, right middle temporal pole DTI FA_p10 (*ρ* = 0.542, *q* = 0.0022), left parahippocampal MAP RTOP_p10 (*ρ* = 0.541, *q* = 0.0022), and left parahippocampal MAP RTPP_p10 (*ρ* = 0.539, *q* = 0.0022) showed positive associations with MoCA scores. Right anterior cingulate DKI MD_mean was negatively correlated with MoCA (*ρ* = −0.541, *q* = 0.0022). Collectively, these findings indicate that gray matter microstructural alterations in the anterior cingulate cortex, parahippocampus, temporal pole, and supramarginal gyrus are closely associated with global cognitive performance. In contrast, no diffusion metric showed a statistically significant association with HAMD-21 scores after FDR correction; therefore, no corresponding correlation plots were generated.

**Figure 2:**
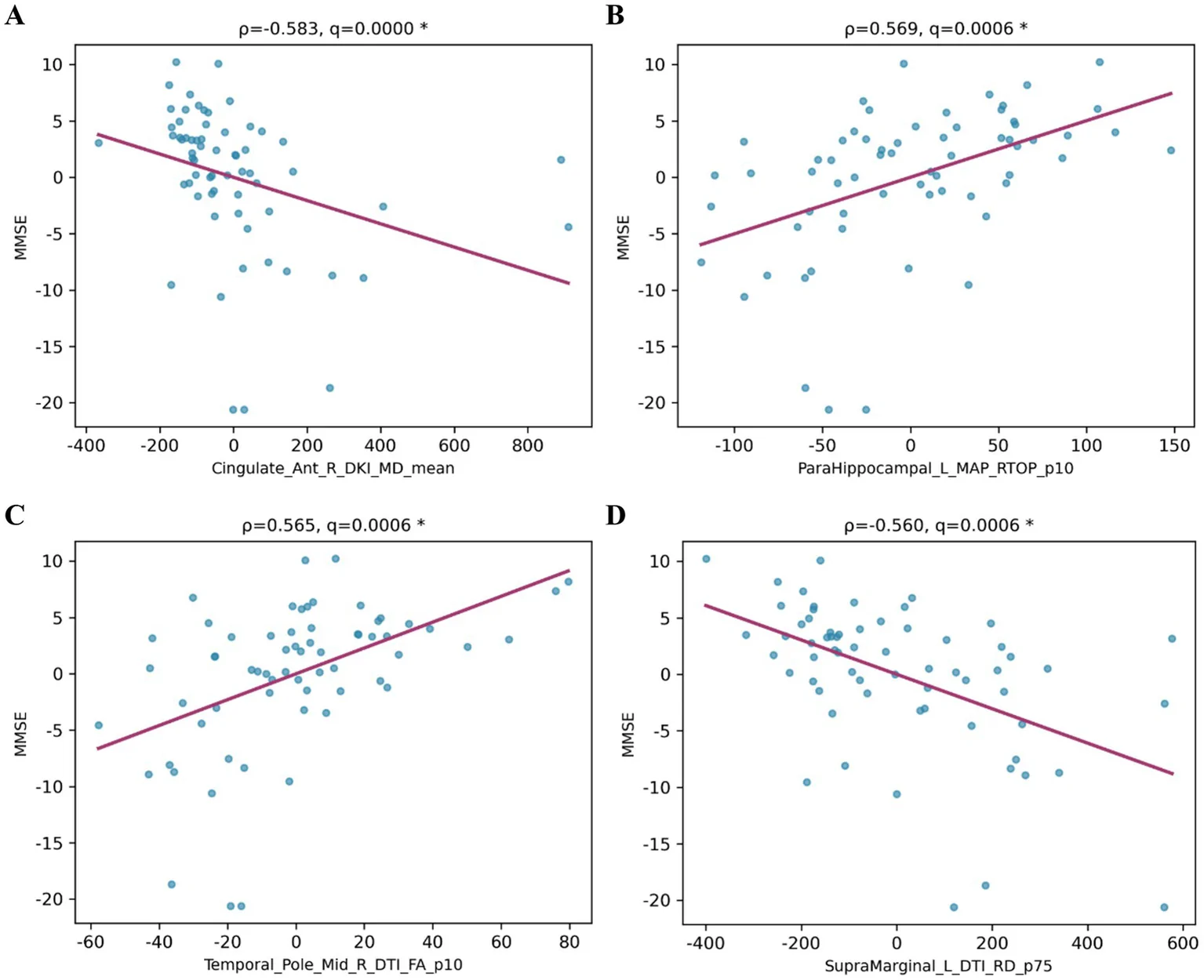
Residual scatter plots with fitted trend lines showing top four significant partial Spearman correlations between multimodal diffusion metrics and MMSE scores. All variables represent residuals after adjustment for age, sex and years of education to eliminate confounding effects. ρ = partial Spearman correlation coefficient; q = FDR-BH corrected *p*-value; * indicates *q* < 0.05. **(A)** Right anterior cingulate DKI MD_mean vs. MMSE; **(B)** Left parahippocampal MAP RTOP_p10 vs. MMSE; **(C)** Right middle temporal pole DTI FA_p10 vs. MMSE; **(D)** Left supramarginal gyrus DTI RD_p75 vs. MMSE.

**Figure 3:**
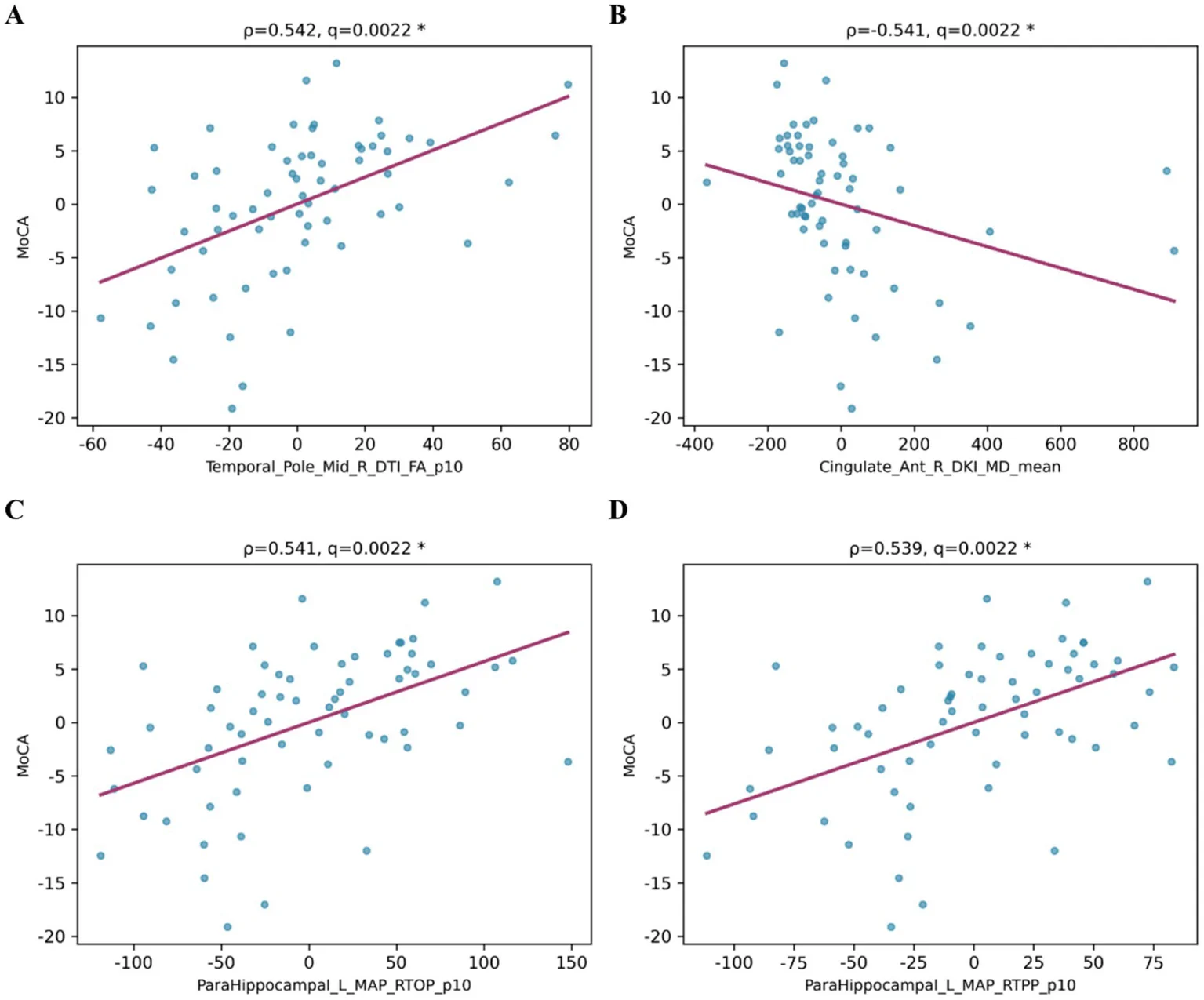
Residual scatter plots with fitted trend lines showing top four significant partial Spearman correlations between multimodal diffusion metrics and MoCA scores. All variables represent residuals after adjustment for age, sex and years of education to eliminate confounding effects. ρ = partial Spearman correlation coefficient; q = FDR-BH corrected *p*-value; * indicates q < 0.05. **(A)** Temporal_Pole_Mid_R_DTI_FA_p10 vs. MoCA; **(B)** Cingulate_Ant_R_DKI_MD_mean vs. MoCA; **(C)** ParaHippocampal_L_MAP_RTOP_p10 vs. MoCA; **(D)** ParaHippocampal_L_MAP_RTPP_p10 vs. MoCA.

## Discussion

The primary objective of this study was to examine the association between specific multimodal diffusion imaging markers and PSD in patients with acute ischemic stroke, with the goal of identifying imaging correlates that could support early detection. By integrating DTI, DKI, MAP and NODDI multi-modal diffusion parameters alongside clinical assessment data from stroke patients and HC, we sought to address gaps in understanding of PSD’s neurobiological mechanisms and to provide preliminary evidence for objective imaging diagnostic indicators.

Partial Spearman rank correlations adjusted for age, sex, and years of education were performed for all FDR-screened significant gray matter diffusion metrics derived from the four diffusion models. Anterior cingulate DKI MD showed significant negative correlations with MMSE and MoCA scores, whereas parahippocampal MAP RTOP and temporal pole DTI FA were positively correlated with cognitive performance. These findings suggest that microstructural alterations in limbic and temporoparietal regions are closely associated with impairments in global cognitive function. Notably, no imaging parameter remained significantly associated with HAMD-21 scores after FDR correction, suggesting that the identified gray matter microstructural abnormalities may be more closely related to cognitive impairment than to depressive symptom severity.

Compared with HC, both PSD and non-PSD stroke patients exhibited significant microstructural abnormalities in the amygdala, middle orbital frontal gyrus, and posterior cingulate gyrus. These regions are key components of neural circuits involved in emotional and cognitive regulation. The posterior cingulate gyrus contributes to the integration of emotional memory and cognitive information, and structural abnormalities in this region may disrupt the interaction between emotional processing and cognitive evaluation (4). The middle orbital frontal gyrus is involved in executive control and emotional decision-making, and its dysfunction may impair the coordination of cognitive judgment and emotional responses (18). The amygdala serves as a central hub for processing negative emotions, including fear and sadness, and microstructural abnormalities in this region may contribute to dysregulated emotional reactivity (19). Prior research has also demonstrated the involvement of the frontal lobe in post-stroke brain alterations (12). Although the specific abnormal regions identified in previous studies differ from those observed in the present study, these findings collectively support an important role for frontal neutral circuits in post-stroke emotional and cognitive dysfunction. Our study further implicate the amygdala, middle orbital frontal gyrus, and posterior cingulate gyrus as potential neural substrates associated with PSD.

Direct comparisons between PSD and non-PSD patients revealed additional microstructural abnormalities in the inferior parietal lobule, middle orbital frontal gyrus, amygdala, and posterior cingulate gyrus. These interconnected regions are involved in emotion regulation, cognitive integration, and emotional memory processing, suggesting that their disruption may contribute to the neurobiological mechanisms underlying PSD. The ROC analyses of the pre-screened significant multimodal diffusion biomarkers further demonstrated their discriminative potential. In the HC versus non-PSD comparison, frontal and limbic DTI/DKI metrics yielded AUC values ranging from 0.890 to 0.916. In the HC versus PSD comparison, multiple NODDI-derived metrics in the putamen and hippocampus showed good diagnostic performance, with AUC values ranging from 0.832 to 0.916. For the discrimination between PSD and non-PSD patients, the left putamen NODDI ODI achieved the highest AUC among the four diffusion models, suggesting that alterations in putaminal neurite organization, in addition to temporolimbic metrics, may be particularly relevant to the presence of PSD.

The inferior parietal lobule is involved in semantic processing, emotional memory integration, and attention control; microstructural abnormalities in this region may interfere with the processing of emotional cues and contribute to depressive symptoms (20). The middle orbital frontal gyrus contributes to the balance between executive control and emotional reactivity, and its dysfunction may reduce the capacity to cope with stroke-related stress (18). The amygdala plays a central role in the recognition and modulation of negative emotions, and microstructural alterations may contribute to sustained emotional hyperreactivity (19). The posterior cingulate gyrus is involved in self-referential processing and the integration of cognitive and emotional information, and abnormalities in this region may be associated with increased negative self-referential processing and feelings of hopelessness (4). Collectively, microstructural abnormalities across these interconnected regions may disrupt neural communication, impair neuroplasticity, and alter the coordination of large-scale brain networks, potentially contributing to the development or exacerbation of depressive symptoms after stroke (21). Our findings are consistent with previous studies of major depressive disorder highlighting abnormalities in frontal-limbic circuits (13). However, the present study further identifies gray matter abnormalities in the inferior parietal lobule and temporolimbic regions that may be particularly relevant to depression following stroke.

Our findings also corroborate and extend previous multimodal diffusion studies of PSD. Shen et al. demonstrated that DKI can detect microstructural changes alterations that may not be captured by conventional DTI by modeling non-Gaussian water diffusion (22). Although their study focused on white matter, whereas the present study investigated gray matter using DTI, DKI, MAP and NODDI simultaneously, both studies support the ability of DKI to characterize tissue heterogeneity. In the present study, however, DKI showed lower diagnostic performance than MAP and NODDI. Lu et al. (23) reported favorable classification performance of NODDI for PSD, and further showed that combining multimodal imaging approaches could improve diagnostic accuracy. Consistent with these findings, our ROC analyses demonstrated reliable discriminative performance of temporolimbic NODDI metrics. Lu et al. (23) also reported that individual DTI parameters had limited ability to discriminate among the three groups, which is consistent with our findings in gray matter. Deng et al. (24) identified reduced FA in white matter tracts and demonstrated its association with HAMD-21 scores using tractography. Beyond stroke populations, Falangola et al. (25) were the first to apply diffusional kurtosis imaging to patients with mild cognitive impairment and Alzheimer’s disease, reporting that non-Gaussian diffusion metrics detect early microstructural damage with AUCs of 0.80–0.90 for discriminating between subject groups. Their observation that non-Gaussian metrics are sensitive to cognition-related microstructural injury is consistent with our finding that the diffusion measures most strongly associated with clinical status were linked to cognitive performance rather than to depression severity. Although the present study did not perform white matter fiber tracking, our partial correlation analyses identified significant associations between limbic diffusion metrics and cognitive performance, further supporting a relationship between gray matter microstructural alterations and post-stroke neuropsychological function.

Several limitations should be acknowledged. First, this was a single-center study with a relatively small sample size, comprising 41 stroke patients and 23 healthy controls. Because ROI screening and ROC analyses were performed using the same dataset without internal or external validation, the reported AUC values and effect sizes should be regarded as exploratory and may be subject to optimism bias. Therefore, these findings require independent validation in larger, multicenter cohorts before any clinical application can be considered. Second, this study included only patients with acute ischemic stroke and excluded those with hemorrhagic stroke, which limits the generalizability of the findings to the broader stroke population. Third, although age, sex and years of education were adjusted for in all statistical analyses, other potentially important confounders, including vascular risk factors, infarct location, infarct volume and stroke severity, were not fully accounted for. Therefore, the observed intergroup differences cannot be attributed exclusively to PSD. In particular, the lack of adjustment for infarct location warrants caution when interpreting group differences in regional gray matter microstructure. Third, infarct volume was not systematically quantified in the present study, and future studies with standardized volumetric assessment are warranted to determine whether lesion burden contributes to the observed microstructural alterations in limbic regions. Finally, mood assessments were performed only 2 weeks after stroke onset, without longitudinal follow-up. The cross-sectional design precludes determination of the temporal or causal relationship between gray matter microstructural alterations and the development of PSD. Longitudinal studies incorporating repeated imaging and clinical assessments from the acute stage of stroke to the development or resolution of PSD are needed to better establish temporal relationships and potential causal mechanisms. Integrating DTI, DKI, MAP, and NODDI with fMRI and perfusion imaging may provide a more comprehensive characterization of PSD-related structural and functional brain remodeling.

Future research should address these limitations by recruiting larger, mixed cohorts that include both ischemic and hemorrhagic stroke and by adopting longitudinal observation designs. Integrating multiple diffusion models with functional and perfusion MRI may provide a more comprehensive characterization of the microstructural and functional alterations associated with PSD. investigating whether these multimodal diffusion biomarkers can predict response to antidepressant treatment may facilitate the development of individualized treatment strategies. Another limitation is that we relied on visual imaging screening to exclude large overt infarcts overlying ROIs, without voxel-wise lesion masking. Although no target ROI was directly covered by macroscopic large infarcts, subtle micro-damage within ROIs cannot be fully ruled out and may confound diffusion-derived quantitative metrics.

## Conclusion

Multimodal diffusion imaging integrating DTI, DKI, MAP and NODDI can detect gray matter microstructural abnormalities within emotion-cognition circuits, particularly in the amygdala, posterior cingulate gyrus, and inferior parietal lobule, across healthy controls and stroke subgroups. Diffusion-derived metrics in these limbic and temporoparietal regions were significantly associated with cognitive performance and demonstrated moderate discriminative performance among HC, non-PSD, and PSD groups in this exploratory analysis. When combined with clinical neuropsychological assessments, these complementary diffusion models may provide preliminary insights into the neural substrates of post-stroke depression, but their potential utility for early identification, monitoring, and personalized management requires independent validation in larger cohorts.

## Data Availability

The raw data supporting the conclusions of this article will be made available by the authors, without undue reservation.
