# Supplementary material for: Comparative evaluation of diffusion models for detecting gray matter microstructural alterations in post-stroke depression patients
Source: Front Neurol. 2026 Sep 30;17:1831557. doi: 10.3389/fneur.2026.1831557 (PMC13661199; doi:10.3389/fneur.2026.1831557)
Supplement: Supplementary file 1 [file Data_Sheet_1.DOCX]

**Model Fitting Method:**

I. **DTI (Diffusion Tensor Imaging)**

- **Signal Model & Fitting Formula:**
  The diffusion tensor eigenvalues ($\lambda_{1}$,$\lambda_{2}$,$\lambda_{3}$) were derived from the tensor matrix to obtain the standard parametric maps.
  - Axial Diffusivity (AD) =$\lambda_{1}$
  - Radial Diffusivity (RD) =$\left( \lambda_{2}+\lambda_{3} \right)/2$
  - Mean Diffusivity (MD) =$\left( \lambda_{1}+\lambda_{2}+\lambda_{3} \right)/3$
  - Fractional Anisotropy (FA) =$\sqrt{\frac{1}{2}}\frac{\sqrt{\left( \lambda_{1}-\lambda_{2} \right)^{2}+\left( \lambda_{1}-\lambda_{3} \right)^{2}+\left( \lambda_{2}-\lambda_{3} \right)^{2}}}{\sqrt{\lambda_{1}^{2}+\lambda_{2}^{2}+\lambda_{3}^{2}}}$

II. **DKI (Diffusion Kurtosis Imaging)**

- **Signal Model & Fitting Formula:**
  To characterize non-Gaussian diffusion, the DKI model extends the DTI signal equation by incorporating the excess kurtosis ($K$):

$$S\left( b \right)=S\left( 0 \right)\exp\left( -b\cdot ADC_{DKI}+\frac{1}{6}b^{2}\cdot ADC_{DKI}^{2}\cdot K \right)$$

The mean kurtosis (MK) was calculated as the average of directional kurtosis ($K_{i}$) across all $n$ diffusion encoding directions:

$$MK=\left( \frac{1}{n} \right)\sum_{i=1}^{n} (\left. K\left. ) \right._{i} \right.$$

III. **MAP-MRI (Mean Apparent Propagator)**

- **Signal Model & Fitting Framework:**
  Instead of a single equation, MAP-MRI models the three-dimensional diffusion propagator $P\left( R \right)$in q-space. The propagator $P\left( R \right)$ indicates the likelihood of particles undergoing a net displacement$R$. The key q-space indices were calculated using the following integrals of the normalized signal $E\left( q \right)$:
  - Return-to-Origin Probability (RTOP) = $\int_{R^{3}} E\left( q \right)\text{ }dq$
  - Return-to-Axis Probability (RTAP) = $\int_{R^{2}} E\left( q_{\perp} \right)\text{ }dq_{\perp}$
  - Return-to-Plane Probability (RTPP) = $\int_{R} E\left( q_{\parallel} \right)\text{ }dq_{\parallel}$
  - Mean Squared Displacement (MSD) = $\int_{R^{3}} P\left( R \right)R^{2}\text{ }d^{3}R$
  - Q-space Inverse Variance (QIV): $QIV^{-1}=\int_{R^{3}} E\left( q \right)q^{2}\text{ }d^{3}q$
  - Non-Gaussianity (NG) = $\sin\theta\cdot PG$, quantifying the dissimilarity between the true propagator $P\left( t \right)$ and its Gaussian part $G\left( t \right)$.

IV. **NODDI (Neurite Orientation Dispersion and Density Imaging)**

- **Signal Model & Fitting Formula:**
  NODDI is a three-compartment biophysical model. The total normalized diffusion signal ($E$) is modeled as the sum of the intracellular, extracellular, and isotropic compartments:

$$E=\left( 1-V_{iso} \right)\left( V_{ic}E_{ic}\left( ODI \right)+\left( 1-V_{ic} \right)\cdot E_{ec} \right)+V_{iso}\cdot E_{iso}$$

where $V_{ic}$ is the intracellular volume fraction, $V_{iso}$ is the isotropic volume fraction, and $ODI$ (Orientation Dispersion Index) characterizes the angular variation of neurites. $E_{ic}$, $E_{ec}$, and $E_{iso}$ represent the signal contributions from the intracellular (hindered), extracellular, and isotropic (free water) compartments, respectively.

All diffusion weighted model fittings (DTI, DKI, MAP MRI, NODDI) in this study were processed with NeuDiLab software (version 1.0, Chengdu Zhongying Medical Technology Co., Ltd., China). This graphical post processing tool directly invokes open source DIPY (and AMICO for NODDI) Python libraries for underlying model reconstruction. All fitting algorithms, physiological constraints and regularization hyper parameters adopted the native default configurations of DIPY/AMICO; no manual modification to any fitting parameters was performed in our analysis.

The key default fitting settings from DIPY/AMICO are summarized belowDIPY：

I. DTI: Weighted least squares (WLS) fitting; tensor eigenvalues were constrained to non negative values.
II. DKI: Weighted least squares (WLS) fitting; physical bounds applied for diffusivity and kurtosis metrics (min_kurtosis = -3/7, max_kurtosis = 10).
III. MAP MRI: Laplacian regularization enabled (laplacian_regularization=True), positivity constraint for propagator (positivity_constraint=True); generalized cross validation (GCV) was used to automatically determine regularization weighting, radial order = 6.
IV. NODDI (AMICO backend): Fixed intrinsic diffusivity constants: parallel intra neurite diffusivity d||=1.7*10^-3mm^2/s, isotropic diffusivity diso=3.0*10^-3mm^2/s; volume fraction parameters constrained between 0 1; Watson distribution for neurite orientation dispersion.
